# TSG-6 expression distinguishes rejected and non-rejected human lung transplants: a retrospective study

**DOI:** 10.3389/fimmu.2025.1685251

**Published:** 2025-12-04

**Authors:** Mohammad Afzal Khan, Dong ge Li, Ashling L. Zhang, Alexander S. Krupnick, Christine L. Lau

**Affiliations:** Department of Surgery, University of Maryland, Baltimore, MD, United States

**Keywords:** TSG-6 (Tumor Necrosis Factor-Stimulated Gene-6), inflammation, immune tolerance, acute rejection, lung transplantation

## Abstract

Lung transplantation (LTx) remains the definitive treatment for select end-stage pulmonary diseases, yet early graft failure due to acute rejection continues to compromise long-term outcomes. Tissue injury and insufficient reparative responses in the immediate post-transplant period contribute to this vulnerability. Tumor Necrosis Factor-Stimulated Gene-6 (TSG-6) is a multifunctional anti-inflammatory and tissue-protective protein known to facilitate resolution of inflammation and extracellular matrix remodeling, but its role in human LTx remains undefined. In this retrospective study, we examined TSG-6 expression in transbronchial biopsies from two matched cohorts of LTx recipients—those with histopathologically confirmed acute rejection (n = 6) and those without rejection (n = 6)—within the first postoperative month. Immunofluorescence analysis revealed significantly elevated TSG-6 expression in non-rejected grafts across two metrics: whole-biopsy staining intensity (Mean 11 ± 1.6 units vs 2.35 ± 0.5, p < 0.01) and percentage of TSG-6–positive cells (~90% vs. ~30%, p < 0.01). Scatter plot visualization confirmed a clear separation between groups, suggesting that elevated TSG-6 expression—both in total tissue and per-cell prevalence—is strongly associated with the absence of acute rejection and may reflect a reparative immune phenotype. Despite the limited sample size, the consistency and magnitude of the effect (Cohen’s d ≈ 4.7) underscore the biological relevance of TSG-6 in early graft stability. Our data establish a novel link between increased TSG-6 expression and diminished acute rejection in human lung allografts, suggesting that TSG-6 may actively modulate alloimmune responses and serve as both a marker of graft stability and a candidate for therapeutic intervention.

## Background

Lung transplantation (LTx) remains a life-saving intervention for patients with end-stage pulmonary diseases (1). However, one of the most critical challenges following the procedure is the risk of acute rejection, particularly within the first month after surgery (2). Inflammation is a central feature of tissue injury following transplantation and plays a pivotal role in both early and late allograft dysfunction (3–7). Ischemia-reperfusion injury (IRI) triggers a sterile inflammatory response characterized by oxidative stress, activation of alveolar macrophages, endothelial injury, and the release of pro-inflammatory cytokines such as TNF-α, IL-1β, and IL-6 (8). These events contribute to tissue damage, increased vascular permeability, and impaired gas exchange—a condition that often present clinically as primary graft dysfunction (9–13). Understanding the molecular mediators and cellular contributors involved in post-transplant lung inflammation is essential for developing targeted therapies to preserve graft integrity and improve transplant outcomes. Tumor Necrosis Factor-Stimulated Gene-6 (TSG-6) is a multifunctional protein that plays a pivotal role in regulating immune responses, promoting tissue repair, and limiting fibrosis (14, 15). TSG-6—secreted primarily by stem cells, fibroblasts, and to some extent by other immune cells—exerts potent anti-inflammatory effects by modulating the activity of immune cells such as M2-polarized macrophages and dendritic cells, reducing the production of pro-inflammatory cytokines, and enhancing the expression of immunoregulatory molecules (16, 17). TSG-6 is minimally expressed under physiological conditions but is rapidly upregulated in response to inflammation and tissue injury—two hallmarks of acute allograft rejection—as reported in preclinical studies of wound healing and transplantation; however, its clinical significance remains undefined, and its short-lived expression may be insufficient to counteract sustained alloimmune inflammation, limiting its capacity to fully control tissue repair in the context of acute rejection (15, 16, 18–21). Thus, measuring TSG-6 levels—via tissue biopsy—could serve as an early indicator of immune tolerance before irreversible tissue damage occurs, which signifies its role as a potential biomarker for identifying and staging the phases of rejection following lung transplantation.

## Method

### Study design

To investigate the expression of TSG-6 in lung transplant recipients, we analyzed existing, de-identified transbronchial biopsies (TBBs) collected one month after lung transplantation from recipients with histologically confirmed rejection (n = 6) and those with stable graft function and no evidence of rejection (n = 6). Specimens were provided by the University of Maryland School of Medicine’s and Greenebaum Comprehensive Cancer Center’s Pathology Biorepository Shared Services, under approved IRB protocol HP-00113750. The biopsy slides were evaluated for TSG-6 expression using immunohistochemistry in the laboratory.

### Immunofluorescence imaging

To examine the tissue expression of TSG-6 in TBBs, both sample groups were immunostained for TSG-6 expression. In brief, paraffin-fixed TBB blocks were sectioned at 5 μm thickness using a microtome, and the sections were mounted on Superfrost Plus slides (Fisher Scientific) for immunofluorescence staining, as previously described (6). Next, Sections were first subjected to antigen retrieval by incubating them with a working solution of Proteinase K (DAKO, Cat. #S3020) for 10 minutes at room temperature. Following this, non-specific binding was initially blocked by incubating the sections with 10% donkey serum for 60 minutes. The tissues were then fixed using a 1:1 methanol/acetone solution for 10 minutes at −20 °C. After fixation and washing, a second blocking step was performed by incubating the slides with 10% donkey serum for 30 minutes to further reduce non-specific antibody binding (22). Subsequently, the slides were incubated overnight at 4 °C with rabbit anti-human TSG-6 primary antibodies (LSBio Cat#LS-C801527). Following overnight incubation, the slides were washed and incubated for 1 hour with Rhodamine Red donkey anti-rabbit IgG secondary antibodies (Cat# 711-296-152, Jackson ImmunoResearch, USA). After secondary antibody incubation, the slides were thoroughly washed and mounted with ProLong Gold antifade reagent containing DAPI (Thermo Fisher Scientific, Cat# P36931) before coverslipping. Immunofluorescence analysis was performed by acquiring images from 15–25 random high-powered fields per biopsy using the Leica DM6 microscope imaging system (Leica Microsystems, USA).

### Image acquisition and data analysis

TSG-6 expression in whole biopsy sections and the percentage of TSG-6–positive cells per sample were quantified using ImageJ software, which applies image threshold scaling to measure the fluorescent intensity of Rhodamine-labeled TSG-6 within tissue compartments; DAPI staining was used to identify and count nuclei, enabling accurate normalization of TSG-6–positive cells relative to total cell number (23–26). GraphPad™ Prism software was used to compare TSG-6 expression between lung transplant biopsies with and without rejection. Due to the apparent non-normal distribution and independent sample structure, a Mann-Whitney U test was applied to assess the significance of this difference. To confirm the appropriateness of non-parametric testing, we performed Shapiro-Wilk tests for normality on both groups. The results indicated significant deviation from normality (No Rejection: p = 0.00004; Rejection: p < 0.00001), thereby justifying the use of the Mann-Whitney U test for group comparisons. A p-value < 0.05 was considered statistically significant for all tests. We also calculated Cohen’s d to quantify the magnitude of difference in TSG-6 expression between rejected and non-rejected lung allografts, providing an objective measure of effect size that supports the biological relevance of our findings despite the limited sample size.

### Imaging controls and signal validation

A secondary antibody–only control was not performed due to limitations in biopsy material; the University of Maryland, Pathology Biorepository Core provided only a single tissue section per slide, which was prioritized for primary antibody staining and quantitative analysis. This limitation precluded the use of adjacent sections for control validation. To minimize nonspecific background and ensure signal specificity, all samples were imaged using the Leica DM6 system under standardized conditions, with an exposure time of 1 second and fluorescence intensity modulation (FIM) set at 10%. Fluorescence signals were consistently localized to expected cellular compartments, and no diffuse or artifactual staining was observed. These imaging parameters support the specificity of TSG-6 detection. While these settings helped minimize visual background and ensured consistent signal acquisition, they do not substitute for antibody specificity controls. Future studies will incorporate formal secondary-only controls to further validate antibody specificity in human lung tissue.

## Results

### Acute rejection is associated with low tissue expression of TSG-6

Biopsies from lung transplant recipients with acute rejection exhibited markedly lower TSG-6 expression compared to those without rejection. Quantitative image acquisition and analysis revealed a significant increase (*p* < 0.05) in TSG-6 staining in the non-rejection group, both at the tissue and cellular levels. Statistical comparisons were performed using the Mann-Whitney U test, and Shapiro-Wilk tests confirmed non-normal data distribution. Effect size was calculated using Cohen’s *d*, yielding a value of ~4.7, indicating an extremely large separation between groups.

Lung tissue is structurally complex and often patchy, particularly in the context of inflammation and rejection, where focal zones of injury or repair can distort localized measurements. To address the inherent spatial heterogeneity of lung transplant biopsies and ensure a biologically accurate representation of TSG-6 distribution, we adapted a dual-quantification approach that measured both total TSG-6 expression across whole biopsy sections and the percentage of TSG-6–positive cells per sample. By analyzing the entire tissue section and normalizing expression to all nucleated (DAPI-positive) cells, we minimized bias from selectively stained regions and captured both the overall burden of TSG-6 and its cellular prevalence. DAPI staining was used to identify nuclei and enable accurate normalization of TSG-6–positive cells relative to total cell number. ImageJ software was used for quantification, applying image threshold scaling to measure the fluorescent intensity of Rhodamine-labeled TSG-6 within tissue compartments.

**Figure 1:**
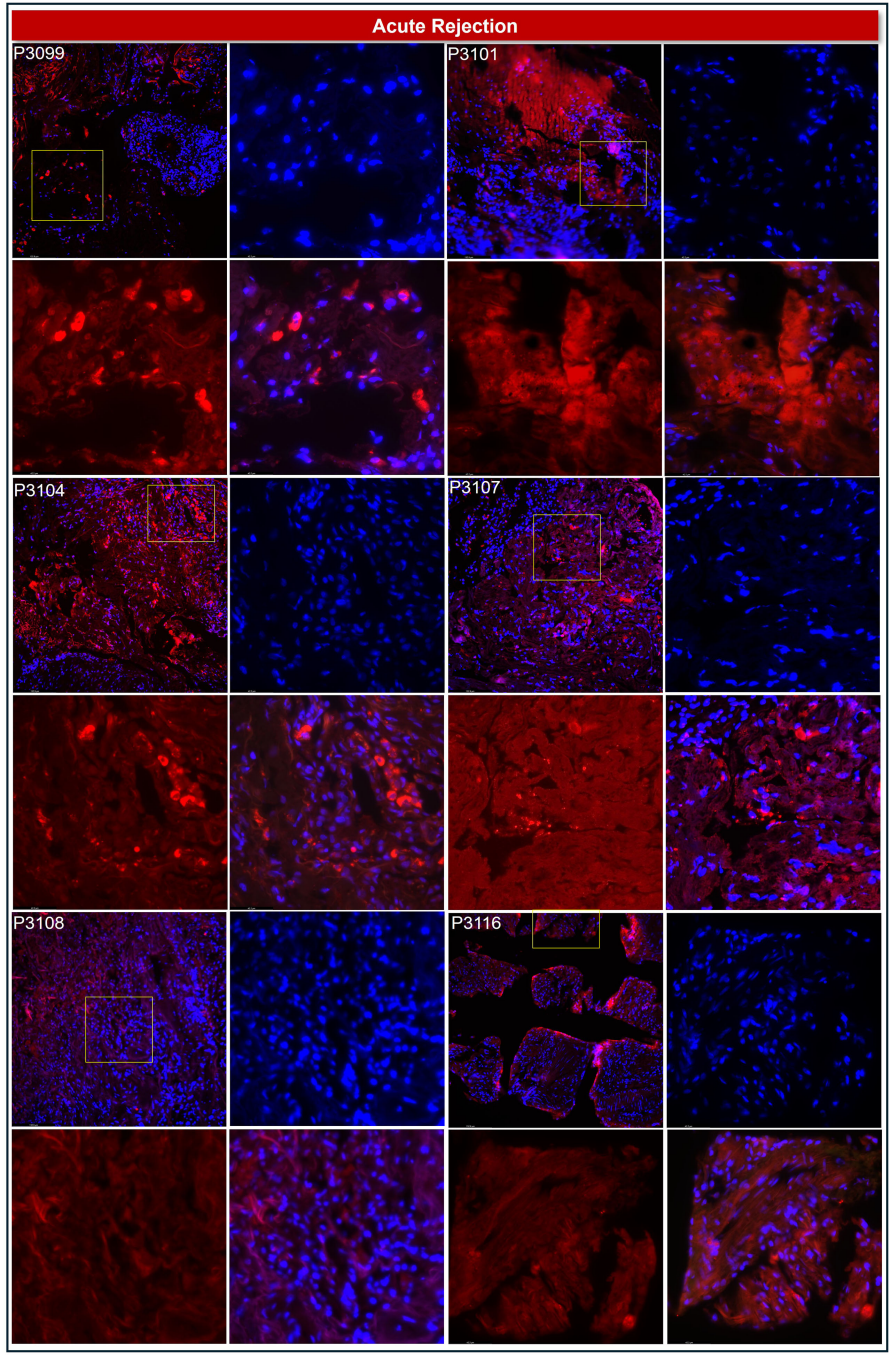
Reduced TSG-6 expression in lung transplant biopsies with acute rejection. Representative immunofluorescence images of lung allograft biopsies from patients diagnosed with acute rejection (P3099, P3101, P3104, P3107, P3108, P3116) show consistently low levels of TSG-6 expression. Each quartet of panels represents imaging at 20× magnification, with insets displaying high-resolution views at 63×. Insets include individual channels for DAPI (nuclei, blue), Rhodamine-conjugated TSG-6 (red), and the merged image.

**Figure 2:**
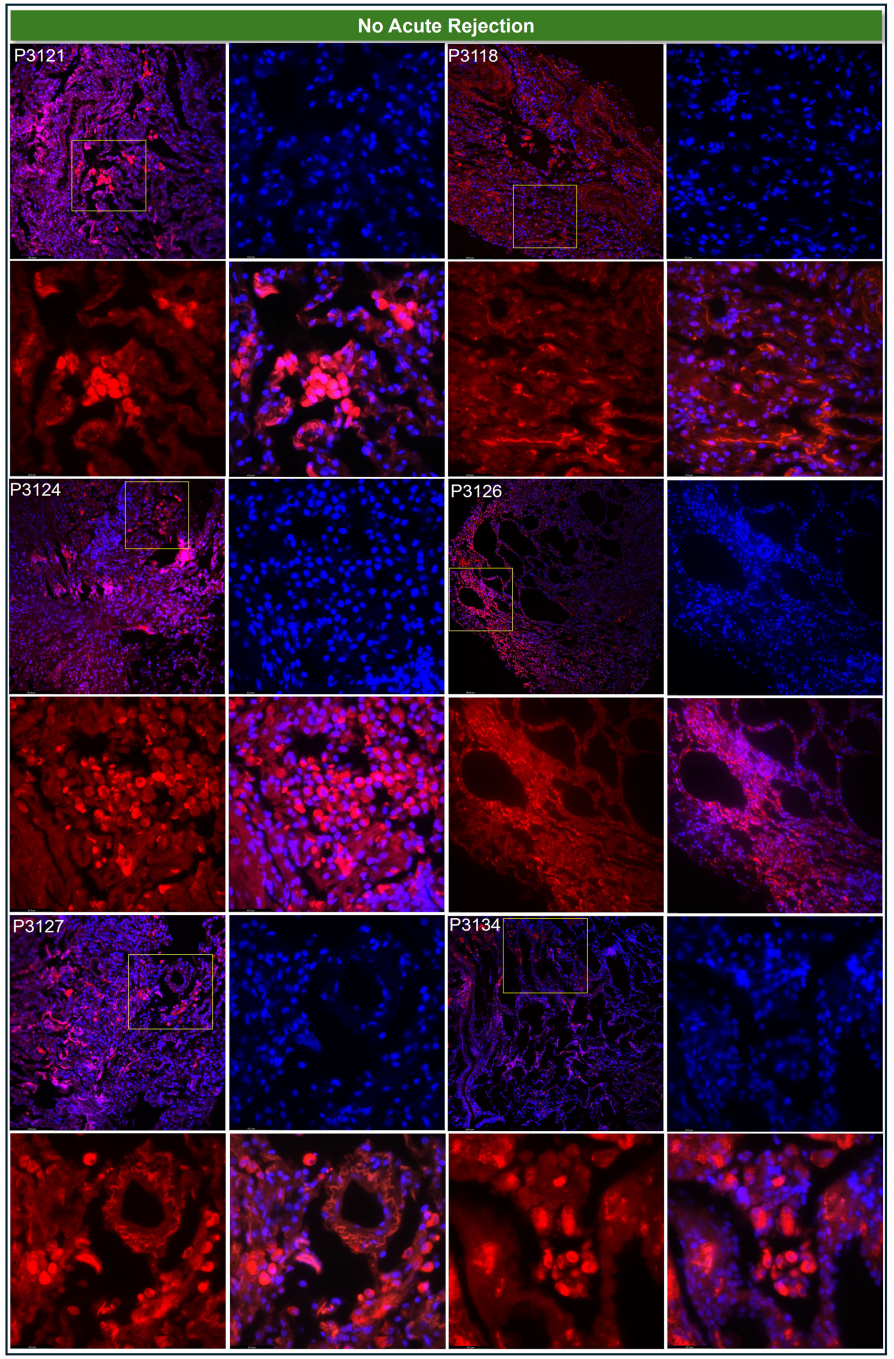
Elevated TSG-6 expression in lung transplant biopsies without acute rejection. Representative immunofluorescence images of lung allograft biopsies from patients without histological evidence of acute rejection (P3121, P3118, P3124, P3126, P3127, P3134). Each quartet of panels shows staining at 20× magnification, with insets displaying high-resolution views at 63×. Insets include individual channels for DAPI (nuclei, blue), Rhodamine-conjugated TSG-6 (red), and the merged image.

This strategy improved interpretability, enabled robust group comparisons, and strengthened the biological relevance of our findings—particularly in a small cohort where consistency across metrics is essential. Qualitative assessment supported these findings: most rejection biopsies (e.g., P3101, P3108, P3116) showed minimal or absent TSG-6 staining, with only focal moderate expression in P3099, P3104, and P3107 (**Figure 1**; **Table 1**). In contrast, non-rejection biopsies such as P3121, P3124, P3127, and P3134 exhibited robust TSG-6 expression, while P3118 and P3126 showed moderate levels (**Figure 2**; **Table 1**).

**Table 1:** Comparative analysis of TSG-6 expression in rejected vs. non-rejected lung allografts.

Patient ID	Rejection (Y/N)	% TSG-6–Positive Cells	TSG-6 intensity (AU)
P3099	Y	39	7751
P3101	Y	0	1222
P3104	Y	55	7574
P3107	Y	45	5174
P3108	Y	19	1991
P3116	Y	10	1830
P3121	N	95	57047
P3118	N	82	15877
P3124	N	90	83123
P3126	N	88	39358
P3127	N	92	54416
P3134	N	96	61475

**Figure 3:**
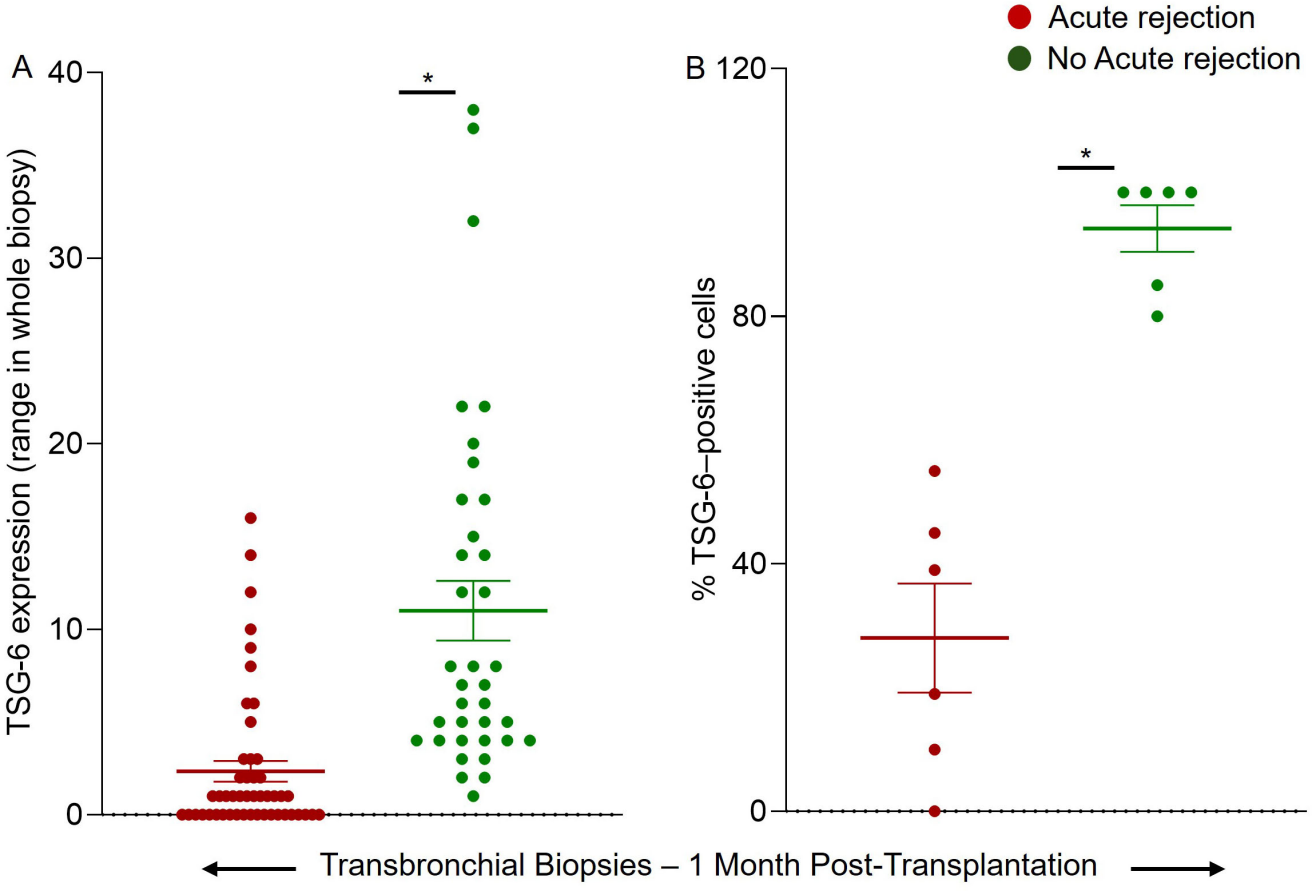
Scatter Plot Analysis of TSG-6 Expression in Biopsies. **(A)** Scatter plot showing TSG-6 expression levels (Mean±SE) in whole biopsy in rejected versus non-rejected groups **(B)** Percentage (%) of TSG-6-positive cells (Mean±SE) in rejected versus non-rejected groups. Each dot represents an individual biopsy, with green indicating non-rejection and red indicating acute rejection. Statistical comparisons were performed using the Mann–Whitney U test (p < 0.05), following confirmation of non-normal distribution via Shapiro–Wilk test. Effect size was estimated using Cohen’s d.

Quantitative data from **Table 1** further support that TSG-6 intensity values in rejected biopsies ranged from 1991 to 7751 arbitrary units (AU), with most samples clustering below 6000 AU, while non-rejected samples showed markedly higher intensities, ranging from 15877 to 83,123 AU (**Table 1**). Whole-biopsy TSG-6 expression showed a clear separation between groups: rejected samples clustered between 0-16 units (Mean 2.35 ± 0.5), while non-rejected samples ranged from 1-38 units (Mean 11 ± 1.6), with several exceeding 30 units (**Figures 3A, B**). Similarly, the percentage of TSG-6–positive cells was significantly higher in the non-rejection group (range: ~80–100%) compared to the rejection group (~0–55%). Group means were ~30% for rejected and ~90% for non-rejected biopsies (n = 6 per group). Scatter plot visualization confirmed this separation, with non-rejected samples exhibiting tightly clustered high expression and rejected samples showing broader variability at lower levels.

Together, these data demonstrate that elevated TSG-6 expression—both in total tissue and per-cell prevalence—is strongly associated with the absence of acute rejection. The magnitude and consistency of this difference, despite the limited sample size, support the biological relevance of TSG-6 as a potential marker of graft stability and reparative immune activity.

## Discussion

Immune tolerance in lung transplantation is orchestrated by a complex network of regulatory molecules that modulate inflammation and tissue repair (27–30). Among these, tumor necrosis factor-stimulated gene-6 (TSG-6) has emerged as a potent immunomodulatory protein that promotes macrophage polarization toward an anti-inflammatory M2 phenotype and facilitates tissue regeneration (17, 31). Translational studies in wound healing and experimental transplantation models have consistently demonstrated the role of TSG-6 in promoting immune tolerance and limiting fibrosis (15, 16, 20, 24, 32–39). However, its clinical relevance in solid organ transplantation remains poorly defined and warrants further investigation.

In this retrospective study, we examined TSG-6 expression in human lung transplant biopsies and found a robust inverse association with acute rejection. Non-rejected biopsies consistently exhibited higher TSG-6 levels, both in total tissue intensity and in the percentage of TSG-6–positive cells. Despite the small sample size (n = 6 per group), the magnitude and consistency of these differences were striking, with a calculated effect size (Cohen’s d ≈ 4.7) indicating an extremely large separation between groups. These findings underscore the biological relevance of TSG-6 as a potential marker of graft stability and a candidate for further mechanistic investigation. This underscores the biological relevance of TSG-6 as a potential marker of graft stability. However, this estimate should be interpreted with caution given the potential for overestimation and outlier influence in small cohorts. The lack of clinical metadata further limits interpretability. Because the transbronchial biopsies were de-identified, we were unable to account for patient demographics, underlying lung disease, immunosuppressive regimens, or histological rejection grades. These variables may influence immune responses and graft outcomes, and their absence precludes subgroup analyses or adjustment for confounders. Another important limitation is that TSG-6 expression was assessed exclusively in tissue biopsies. While this provides direct insight into local graft biology, transbronchial biopsy is an invasive procedure and not feasible for routine longitudinal monitoring. Future studies should evaluate whether TSG-6 levels in less invasive samples—such as blood, bronchoalveolar lavage fluid, or extracellular vesicles—correlate with tissue expression and rejection status, thereby enhancing its translational utility as a biomarker. Finally, while our findings are biologically compelling, they remain correlative. Functional studies are needed to determine whether TSG-6 actively modulates immune responses or merely reflects the inflammatory state of the graft. TSG-6 is a rapidly inducible anti-inflammatory protein secreted by mesenchymal stem cells, fibroblasts, and immune cells in response to tissue injury. Its roles in wound healing, immune modulation, and macrophage polarization toward an M2 phenotype are well documented in preclinical models, where it has been shown to support tissue repair and promote IL-10–mediated anti-inflammatory pathways. Expression of TSG-6 during alloimmune inflammation has been reported in small animal models (15, 19, 20, 31, 39–41), yet its mechanistic role in human lung transplantation remains undefined. Importantly, our study provides the first evidence linking elevated TSG-6 expression with reduced acute rejection in human lung allografts. Non-rejected biopsies exhibited consistently higher TSG-6 levels, suggesting a potential role in graft preservation. Although we did not directly assess immune cell phenotypes or cytokine profiles, these findings raise the possibility that TSG-6 contributes to a reparative immune environment in human lung transplantation—a hypothesis warranting further mechanistic investigation.

Collectively, our findings identify TSG-6 as a promising candidate biomarker for lung allograft rejection. Its distinct expression profile in non-rejected biopsies suggests potential utility in stratifying patients by rejection status and guiding personalized monitoring strategies. The strong inverse association between elevated TSG-6 levels and acute rejection highlights its relevance as a diagnostic marker of graft immune state. While these results are biologically compelling, validation in larger, clinically annotated cohorts will be essential to confirm these observations and determine whether TSG-6 can reliably inform rejection risk or therapeutic decision-making in human lung transplantation. Further investigation is also needed to clarify its mechanistic role in transplant immunobiology and assess its potential as a target for immunomodulatory interventions.

## Data Availability

The raw data supporting the conclusions of this article will be made available by the authors, without undue reservation.
